# Highly Efficient and Stable Novel NanoBiohybrid Catalyst to Avert 3,4-Dihydroxybenzoic Acid Pollutant in Water

**DOI:** 10.1038/srep33572

**Published:** 2016-10-10

**Authors:** Rasel Das, Sharifah Bee Abd Hamid, Mohamad Suffian Mohamad Annuar

**Affiliations:** 1 Nanotechnology and Catalysis Research Center (NANOCAT), University of Malaya, 50603 Kuala Lumpur, Malaysia; 2 Institute of Biological Sciences, Faculty of Science, University of Malaya, 50603 Kuala Lumpur, Malaysia

## Abstract

The present study reported for the first time covalent immobilization of protocatechuate 3,4-dioxygenase (3,4-POD) onto functionalized multi-walled carbon nanotubes (F-MWCNT) for degrading the toxic 3,4-dihydroxybenzoic acid (3,4-DHBA) pollutant in water. The F-MWCNTs had a maximum 3,4-POD loading of 1060 μg/mg. Immobilized 3,4 POD had 44% of relative structural changes to its free configurations. Nevertheless, >90% of relative activity and about 50% of catalytic efficiency were retained to the free enzyme. Immobilized 3,4-POD demonstrated higher alkaline stability and thermostability than the free 3,4-POD. The free and immobilized 3,4-POD lost 82% and 66% of relative activities, respectively after 180 min of incubations at 90 °C. Excellent shelf-life was observed for the immobilized 3,4-POD with residual activity of 56% compared with 41% and 39% of the free 3,4-POD at 4 °C and 25 °C over 30 days storage. Immobilized 3,4-POD showed >60% of catalytic activity retention even after ten-cycle uses, defraying the expenses of free 3,4-POD productions for long term uses. Finally, the immobilized 3,4-POD removed 71% of 3,4-DHBA from water in <4 h, paving its future application for water purification with reduced costs and time.

Industrial food processing waste water effluents account for the 3,4-dihydroxybenzoic acid (3,4-DHBA) concentrations in the environment^1^. This dwindling of the finite fresh water resources, seriously affects the terrestrial, aquatic, and aerial flora and fauna. The 3,4-DHBA has shown contradictory biological effects on the animal and human tissues. Some authors hypothesize the 3,4-DHBA can inhibit chemical-actuated-carcinogenesis of various mouse tissues such as liver, kidney, skin and so on^2^; whereas others have proved that the compound has decreased the level of glutathione – a major cellular antioxidant. It induces oxidative stress; and causes hepatotoxicity, neurotoxicity, tumor productions, and inflammations^3^,^4^. Babich *et al.*^5^ found that the 3,4-DHBA with a concentration from 5 to 25 mM could be significantly toxic for normal human cells and nontoxic to malignant cells. Therefore, effective and inexpensive regulatory tool should be developed to remove the 3,4-DHBA from water.

Several studies employing Fenton^1^, adsorption^6^, O_3_/UV or H_2_O_2_/UV,^7^ and microbial degradation^8^ have been adopted to remove the 3,4-DHBA from water but these methods are less selective, ineffective for dilute solution, time consuming, energy intensive, and generate toxic byproducts^9^. In contrast, the judicial choices of using enzyme for water purification are due to its high selectivity and sensitivity, fast reaction kinetics, fewer byproducts formation, minimal energy consumption and finally benign for the environment as compared with the physical and chemical methods^10^. However, the free enzyme is not stable under mechanical and chemical stresses and difficult to separate from the substrates in a reaction vessel. In order to overcome these hurdles, immobilization of enzymes onto a physical support is a must^11^ for water purification.

The protocatechuate 3,4-dioxygenase (3,4-POD; EC: 1.13.1.3, MW: 700 kDa) is an intradiol cleaving enzyme commonly found in *Pseudomonas putida*^12^ which consists of *α-* and *β*-subunits (*αβ*)_n_, where n = (2–12)^13^. It has a non-heme Fe (III) at the active site that participates in the direct degradation of 3,4-DHBA^14^ to 3-carboxy-cis,cis-muconic acid (CMA) without any byproducts formations^15^. The MA has not shown toxicity effects on normal cells^16^, and is an industrially valuable compound for adipic acid production (2.3 million metric tons/year and expected market value was 6.3 billion pounds in 2012)^17^. A few studies have been attempted to immobilize 3,4-POD onto porous glass^18^,^19^, agarose^20^,^21^ and alginate matrix^21^,^22^. However, the studies were done without proper scrutiny. They have used the crude 3,4-POD extracts^21^,^22^, and uncharacterized immobilized 3,4-POD structures and apparent kinetic details. Hence, these data should be used with caution in understanding the 3,4-POD catalytic efficiency and behavior. No study to date has yet been published or attempted to immobilize the pure 3,4-POD on nanomaterials. Here we used the multi-walled carbon nanotubes (MWCNT) as a nanocarrier for 3,4-POD immobilization because of its low cost, high aspect ratio, and high chemical stability, and thermostability^23^,^24^. In addition, the MWCNTs are stable and inert toward microbial contaminations^25^, which endows the immobilized 3,4-POD with prolonged activity in wastewater treatment plant. In our earlier study, we reported that a hollow CNT could provide frictionless transport of water molecules that compensate the energy costs for water treatment^24^,^26^.

In this study, the 3,4-POD was covalently immobilized onto functionalized (F)-MWCNTs using cross-linker 1-ethyl-3-(3-dimethylaminopropyl)carbodiimide (EDC) reactions^27^,^28^. However, the hydrophobic CNTs become aggregated and precipitated in water^29^ which impede their uses for enzyme immobilization^30^ and water purification^24^. Thus prior to the 3,4-POD immobilization, we functionalized the pristine MWCNTs using H_2_SO_4_ and HNO_3_ (3:1) and obtained a very stable colloidal MWCNT solution for better MWCNT and 3,4-POD interactions. Immobilized 3,4-POD shown higher stability, good catalytic efficiency, recrudescence and reusability; and thus would lead to efficient 3,4-DHBA breakdown in water with reduced cost and time.

## Results and Discussion

### Characterization of MWCNT Functionalities and Water Solubility

**Figure 1:**
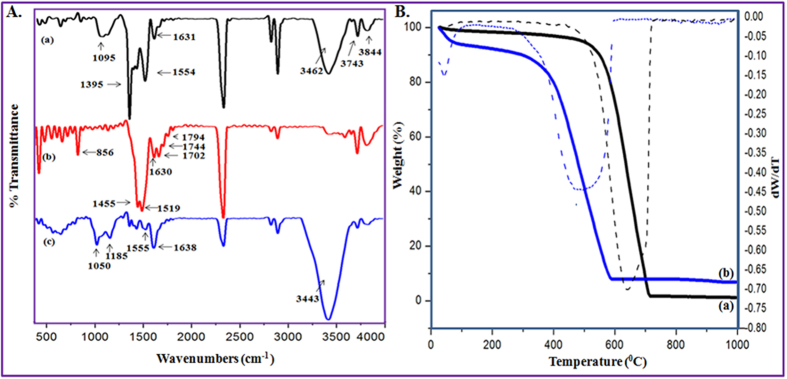
(**A**) ATR-IR spectra of (a) pristine, (b) F-MWCNTs and (c) immobilized 3,4-POD; (**B**) TGA (solid lines) and DTG (dashed lines) plots of (a) pristine and (b) F-MWCNTs, respectively.

The pristine MWCNTs are insoluble in water resulting in decreased optimum enzyme loading^31^. In addition, the inert CNTs may require chemical modifications to covalently attach molecules on its surface^27^. Therefore, MWCNT was treated with H_2_SO_4_/HNO_3_ (3:1) to anchor appropriate functional groups needed for a stable MWCNT colloidal solution. Figure 1(A) displays the attenuated total reflectance infrared (ATR-IR) spectra of pristine (a) and F-MWCNTs (b). Figure 1(Aa) exhibits prominent peaks at 1095, 3462, 3743, and 3844 cm^−1^; suggesting the presence of different forms of -OH groups, which might be generated upon atmospheric amorphous carbon oxidations^32^. Compared with Fig. 1(Aa), some new peaks in Fig. 1(Ab) were observed at 856, 1702; 1744 and 1794 cm^−1^ corresponding to the presence of C-O-O-C, COOH; and C=O^33^,^34^,^35^. Herein the COOH group was significant because of its acting as an ideal attachment point for enzyme immobilization^27^ and improving the F-MWCNT dispersion in water^36^. The functional group helps to induce the reaction of the free amine groups on the surface of enzyme molecule. However, some shifted peaks at 1395, 1554 and 1631; and 1455, 1519 and 1630 cm^−1^ in Fig. 1(Aa,Ab); respectively were also observed because of stretching vibrations of nanotube aromatic rings^33^,^34^,^35^.

Figure 1(B) shows the thermal gravimetric analysis (TGA) and derivative thermogravimetric (DTG) plots of pristine (a) and F-MWCNTs (b). Compared with Fig. 1(Ba), spectrum of Fig. 1(Bb) started first stage decomposition at 100 °C. This was attributed to pyrolytic evolution of water molecules^37^. Second weight loss occurred from 280 °C to 380 °C due to the evaporation of –COOH groups from the F-MWCNT surfaces^38^. Finally, the MWCNTs in Fig. 1(Ba) were burned at 700 °C compared with Fig. 1(Bb) at 580 °C; suggesting purified and functionalized graphitic sheets of Fig. 1(Bb)^39^,^40^. The result is consistent with the aforementioned ATR-IR data as shown in Fig. 1(A). Consequently, F-MWCNTs produced a good and stable colloidal solution even for one month as shown in Supplementary Fig. S1. Therefore, we hypothesized that H_2_SO_4_/HNO_3_ treatment could be a practical method for surface functionalization to bring enzyme towards the reactive surfaces of the F-MWCNTs resulting in better immobilization efficiency.

### Preparation and Optimization of 3,4-POD Loading on F-MWCNTs

**Figure 2:**
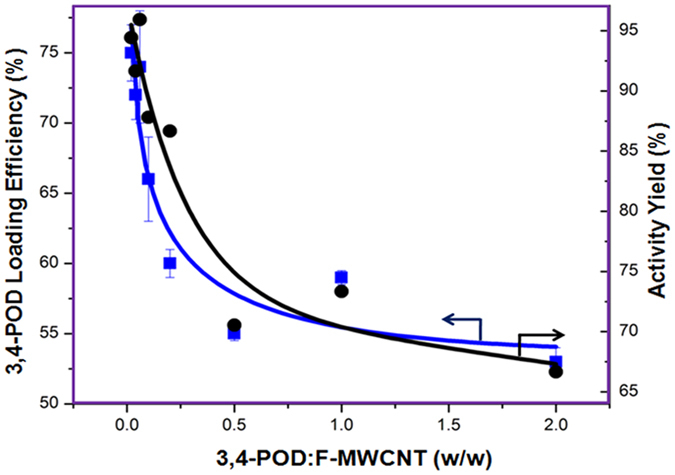
Efficiency of 3,4-POD loadings and activity retention on the F-MWCNTs. Triplicate measurements were taken for all reactions as indicated by error bars.

The covalent method is preferred to the non-covalent in order to develop a stable and robust MWCNT-3,4-POD hybrid system. The coupling buffer (4-(2-hydroxyethyl)-1-piperazineethanesulfonic acid) (HEPES) (50 mM, pH 8.0) was used to maintain the MWCNT colloidal stability and immobilization efficiency^18^,^20^,^22^. Immobilized 3,4-POD with high activity was obtained only after 2 h of incubation as shown in Supplementary Fig. S2 which was a greatly reduced time than the porous glass media (75 and 24 h)^18^,^19^ and agarose matrices (75 and 18 h)^20^. In order to obtain higher 3,4-POD loading efficiency and activity yield, we investigated optimum weight ratio of 3,4-POD (0.02–2.0 mg) to F-MWCNTs (1.0 mg) as shown in Fig. 2. The 3,4-POD loading efficiency onto F-MWCNTs and immobilized 3,4-POD activity yield were calculated using eqs (1) and (2)^41^, respectively.


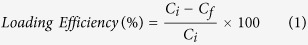


where *C*_*i*_ is the initial enzyme concentration (mg) added to the solution and C_*f*_ is the final enzyme concentration (mg) measured by the Bradford and bicinchoninic acid (BCA) assays in washing solutions.


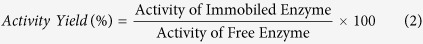


According to the Fig. 2, the maximum (75) and minimum (53%) 3,4-POD loading efficiencies were observed when the lowest and highest concentrations of 3,4-POD were used, respectively. The loading of 1060 μg 3,4-POD/mg on MWCNT (53%) is still considered to be the highest compared with the other immobilized enzymes on CNT^42^,^43^,^44^,^45^,^46^. The yield might be due to the use of F-MWCNT as a water soluble nanocarrier, optimized immobilization reaction conditions, and the nature of free enzyme molecules favorable for attachment. On the other hand, the 3,4-POD activities retained after immobilization ranges from 95% to around 66%; suggesting the MWCNT could be a good support for enzyme immobilization. Lower immobilized 3,4-POD activity at the higher enzyme concentration used was primarily attributed to ‘overcrowding’ effects on F-MWCNT attachment sites of 3,4-POD thus lowering catalytic efficiency towards the 3,4-DHBA^47^. However, both the loading efficiency and retained activity of the 3,4-POD reached a constant value after a ratio of 1.0; suggesting a threshold value after which saturation of reactive site ensured for 3,4-POD attachment. Therefore, the ratio 1.0 was selected for further experiments to avoid inefficient immobilization.

### Characterization of Immobilized 3,4-POD

**Figure 3:**
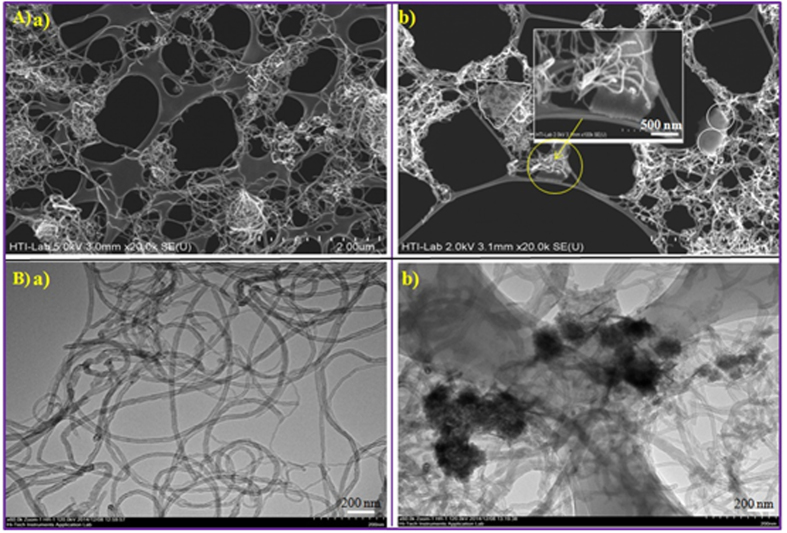
(**A**) SEM and (**B**) TEM images of (a) F-MWCNTs, (b) 3,4-POD immobilized on F-MWCNTs.

**Figure 4:**
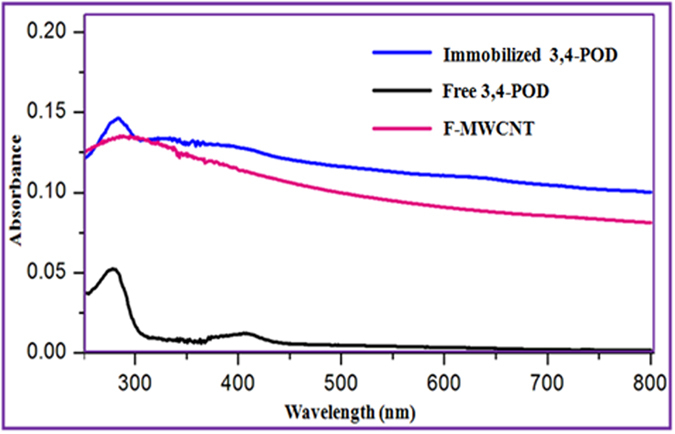
UV-Vis spectra of F-MWCNTs, free and immobilized 3,4-POD solutions.

First, Fig. 1(Ac) displays IR characteristic bands of immobilized 3,4-POD. Compared with Fig. 1(Aa,Ab), unique IR peaks at 1050 and 1185 cm^−1^ in Fig. 1(Ac) resulted from C-N bond formations between the F-MWCNTs and the 3,4-POD^28^,^48^. Meanwhile, the two major unique IR characteristic bands of 3,4-POD at 1555 and 1638 cm^−1^ were observed in Fig. 1(Ac) which were attributed from the 3,4-POD amide II (a blending of major N-H and minor C-N) and amide I (a combination of major C=O and minor C-N), respectively^49^. However, a non-negligible high intense peak at 3443 cm^−1^ in Fig. 1(Ac) might have resulted from either –NH or –OH stretching vibrations^33^,^49^ of the 3,4-POD amino acid side chains. Second, Fig. 3 exhibits field emission scanning electron microscope (FE-SEM) (A) and transmission electron microscope (TEM) (B) observations of the F-MWCNTs (a) and immobilized 3,4-POD (b). According to Fig. 3(Aa,Ba), the F-MWCNTs appeared as clean, fresh, and completely amorphous with metals free threads like MWCNTs; suggesting a good matrix for 3,4-POD attachment. Compared with Fig. 3(Aa,Ba), images in (Ab and Bb) clearly indicated successful immobilization of 3,4-POD onto F-MWCNT. Figure 3(Ab (inset)) reveals the local tips binding domain of the F-MWCNTs for 3,4-POD, since most of the 3,4-POD were anchored onto the F-MWCNT at particular points as shown in Fig. 3(Ab,Bb).This might be due to the -COOH functionalities present at F-MWCNT tip ends. The phenomenon was similar with an earlier observation of Gao and Kyratzis (2008)^27^. They hypothesized that the uniform distribution of enzymes onto F-MWCNTs is inconsistent with the –COOH groups, which could only anchor at MWCNT tip ends or defected sites. The finding was also similar with our recent observations of MWCNT functionalizations^36^. Therefore, we suggested that such local attachment could increase center-to-center distance of 3,4-POD which might help to avoid close packing effects and other unwanted interactions that lead to decreased enzyme activities. Successful localized immobilization of 3,4-POD was also confirmed by the atomic force microscopy (AFM) studies as discussed in Supplementary Fig. S3. The controlled area where the presence of 3,4-POD was not recognized showed smooth mean surface roughness 22 ± 8 nm (Supplementary Fig. S3 (Profile I)) compared with three noticeable hill areas of 77 ± 28 nm in 3,4-POD heights (Supplementary Fig. S3 (Profiles II, III and IV)). Third, Fig. 4 shows the ultraviolet-visible (UV-Vis) spectra of the F-MWCNTs as control, free, and immobilized 3,4-POD solutions. The F-MWCNTs spectrum was shown as a flat line, whereas the immobilized 3,4-POD recorded a peak at 280 nm similar to the free 3,4-POD. Finally, we used circular dichroism (CD) spectroscopy studies to identify the structural variations between the immobilized 3,4-POD and free 3,4-POD as shown in Supplementary Fig. S4. About 44% of relative structural change of the immobilized 3,4-POD was observed which was almost similar to earlier studies performed for other immobilized enzymes^50^,^51^. Conversely, we observed that *α-*helix content was increased from <0.1% (free) to 5.9% (immobilized) 3,4-POD, whereas a decrease in *β*-sheet was observed from 50.04% (free) to 18.1% (immobilized) 3,4-POD. The conformational change of immobilized 3,4-POD might be because of the interactions between free amine groups on the surface of 3,4-POD and –COOH groups of CNT^45^. However, no significant changes were observed for turn (12.5%) and random coils (40%) of free and immobilized 3,4-POD, respectively.

### Effects of pH and Temperature on 3,4-POD Acitivity

**Figure 5:**
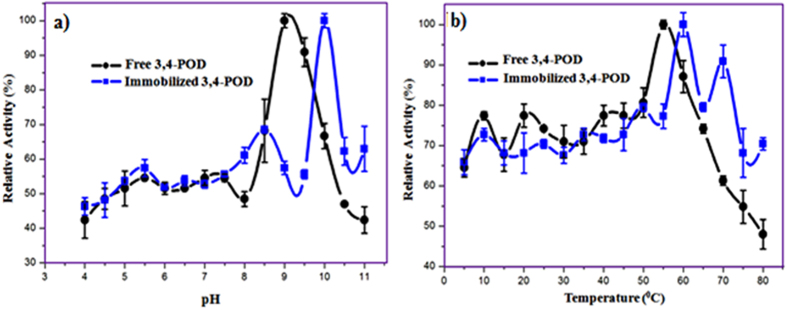
Effects of (**a**) pH and (**b**) temperature on 3,4-POD activities.

Figure 5 shows the effects of pH (a) and temperatures (b) on the free and immobilized 3,4-POD relative activities. It was observed that the free and immobilized 3,4-POD activity profiles followed similar trends up to pH 8.0 and temperature 50 °C, beyond which the activities were varied. The 3,4-POD optimum activities were shifted from pH 9.0 (free) to 10.0 (immobilized) and temperature from 55 °C (free) to 60 °C (immobilized), respectively which was similar to earlier observations^21^. The immobilized 3,4-POD showed significantly higher activities i.e. 63% and 70% than the free 3,4-POD i.e. 42% and 48% (*p* < 0.05) at pH 11.0 and 80 °C, respectively. The improvement of activities against higher alkali and temperatures indicated higher stability of the immobilized 3,4-POD than its free counterpart. The effects were attributed to MWCNT properties and the microenvironmental changes that could affect the conversion of substrate to product^20^. The higher activity of immobilized 3,4-POD at more alkaline pH was suggested to be due to the slower diffusion of 3-CMA into the external solvent phase from the active sites^20^.

### Kinetic Analyses of Free and Immobilized 3,4-POD

The relative activity and all the essential kinetic parameters, which are highlighted in Table 1 were measured from the Michaelis-Menten plots of the free and immobilized 3,4-POD (Supplementary Figs (S5 and S6). The activity retained by the immobilized 3,4-POD was >90%, which was the highest of the activities of immobilized 3,4-POD on porous glass (70% and 90%)^18^,^19^ and agarose (42% and 68%)^18^. This high activity might be due to the nature of support, selection of appropriate immobilization method, optimum immobilized conditions and 3,4-POD source. The higher *K*_*m*_of the immobilized 3,4-POD (2.5×) than that of the free 3,4-POD indicated a reduced 3,4-DHBA binding affinity. Nevertheless, it was still lower than the *K*_*m*_of 3,4 POD immobilized on agarose (15–20 times greater than free enzyme)^20^; suggesting abridged mass transfer limitations for the immobilized 3,4-POD. The result of increased *K*_*m*_ value is also consistent with our earlier CD studies. Increased *α-*helices and decreased *β-*sheets might be the causal effects of about 50% loss of immobilized 3,4-POD overall reaction selectivity (*K*_*cat*_*/K*_*m*_). However, the immobilized 3,4-POD showed a higher turnover number (*K*_*cat*_) and significantly increased reaction rate *V*_*max*_ than the free 3,4-POD (*p* < 0.05). Clearly, the active site residues of the immobilized 3,4-POD were well maintained; suggesting appropriate support of MWCNTs^52^.

### Stability Studies of Free and Immobilized 3,4-POD

**Figure 6:**
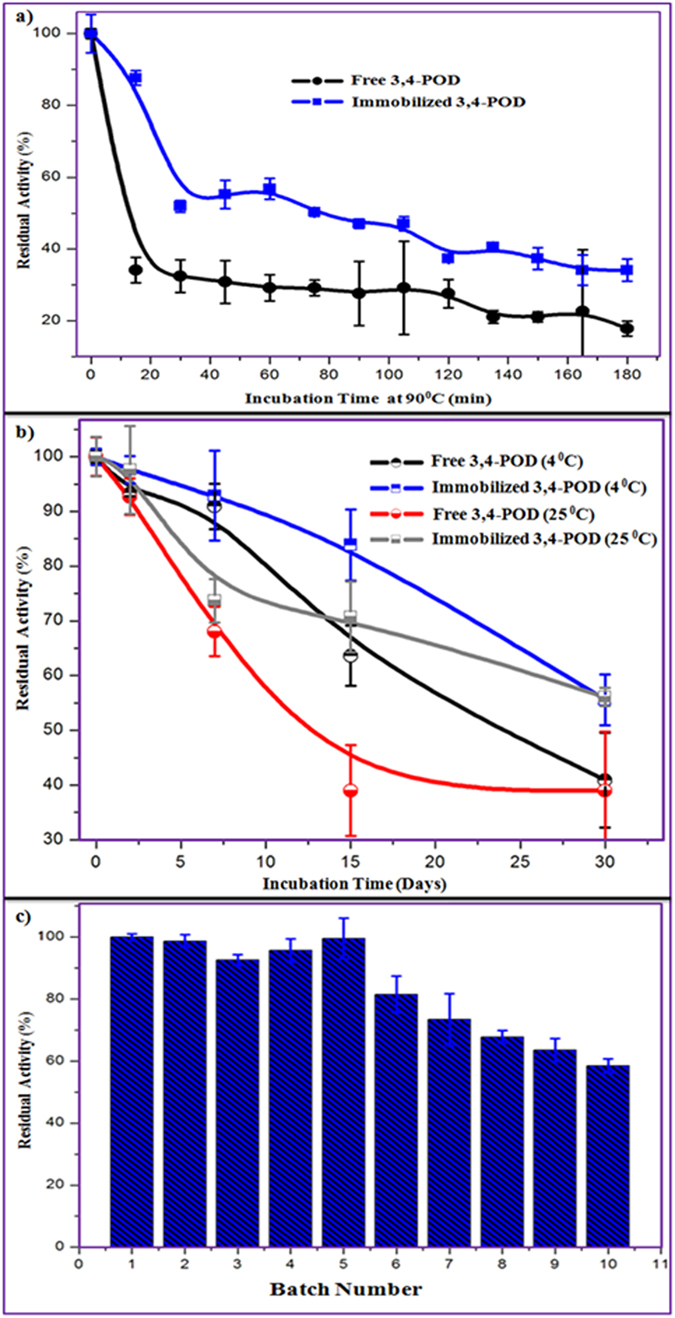
(**a**) Thermostability, (**b**) storage stability of the free and immobilized 3,4-POD; and (**c**) reusability of the immobilized 3,4-POD.

One of the justifications for immobilizing the enzyme onto a support is to intensify its lifespan at extreme conditions. Figure 6 displays the thermostability at 90 °C (a) and storage stability (b) of the free and immobilized 3,4-POD. According to Fig. 6(a), the immobilized 3,4-POD was significantly more stable than the free 3,4-POD (*p* < 0.05). The activity of free 3,4-POD was lost (82%), whereas immobilized 3,4-POD showed (66%) after 180 min incubation. This thermostability might be due to the support mediated conformational changes of 3,4-POD, nature of support, and reduction in molecular mobility^53^. Zaborsky and Ogletree (1972)^20^ found no significant activity changes of the free and 3,4-POD immobilized onto agarose. They reported that the free and immobilized 3,4-POD were inactivated after 60 min and 75 min at 60 °C, respectively^20^. Hence, the immobilized 3,4-POD onto F-MWCNTs may be used to tackle hot industrial effluents.

As revealed by Fig. 6(b), the activities of free 3,4-POD were shown to decrease compared with the immobilized 3,4-POD at 4 °C and 25 °C over 30 days storage. The immobilized 3,4-POD retained 56% of residual activity after 30 days which was 41% and 39% of the free 3,4-POD at 4 °C and 25 °C, respectively (*p* < 0.05). Guzik *et al.*^21^ observed fewer storage stabilities, which were 30% and 10% of the immobilized 3,4-POD onto alginate and agarose after 21 and 28 days, respectively. It suggested that the immobilized 3,4-POD onto F-MWCNTs was significantly resistant to its activity inhibition during longer incubation period than the free 3,4-POD. The chemical bonding between the F-MWCNTs and the 3,4-POD could prevent structural denaturation of the enzyme on long shelf life. Similar hypothesis had been reported for laccase enzyme^54^. The enhancement of overall storage stabilities of the immobilized 3,4-POD onto F-MWCNTs would be advantageous for transportation of the hybrid to be used in remote water purification reactors. Consequently, it can be used as point-of-use (POU) device at point-of-generation (POG) of pollutant effluents. This may help to decrease the labor and materials costs that would make the hybrid commercially feasible.

The recrudescence of the immobilized 3,4-POD is shown in Fig. 6(c). Appropriate physical properties of MWCNTs^24^ allowed us to separate them along with the immobilized 3,4-POD from the reaction mixtures. It would decrease the production cost of 3,4-POD in catalytic applications and subsequently increase the feasibility of the developed hybrid to be used industrially. As shown in Fig. 6(c), >97% (mean) of the immobilized 3,4-POD activity was retained up to five batches uses. After ten cycles, the activity was kept around 60%; suggesting greater operational stability of the immobilized 3,4-POD. Although no reusability data of 3,4-POD was reported by the previous studies^19^,^20^,^21^,^22^, similar data can be comparable to other enzymes immobilized on different supports^48^,^51^.

### Degradation Kinetics of 3,4-DHBA

**Figure 7:**
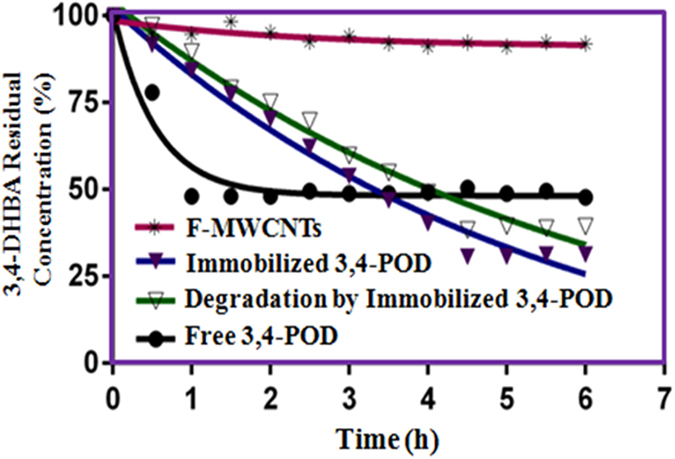
Degradation kinetics of 3,4-DHBA by the optimized free- and immobilized 3,4-POD.

The cytotoxicity of 3,4-DHBA to human cell lines had been suggested to occur at 5.0 mM (*p* ≤ 0.01), whereas nontoxic level was from 1.0 to 2.5 mM^5^. Hence, it is not unreasonable to investigate the removal of 5.0 mM 3,4-DHBA by the immobilized 3,4-POD. Figure 7 shows the removal percentages of 3,4-DHBA in a 6 h batch experiment. Data were fitted to first-order-reaction, and the important kinetic parameters with the removal efficiency (RE) are listed in Table 2. The immobilized 3,4-POD showed a slower 3,4-DHBA removal rate (t_*1/2*_: 3.81 h) than the free enzyme (t_*1/2*_: 0.37 h). The phenomenon is consistent with our earlier *K*_*m*_and *K*_*cat*_*/K*_*m*_ data. It was suggested to be due to the curtailment of 3,4-DHBA diffusion to the 3,4-POD active sites, low molecular flexibility and conformational changes upon 3,4-POD immobilization onto F-MWCNT surfaces^53^. Another hypothesis could be the 3-CMA, which may create steric blockage once its releases from the external milieu of 3,4-POD active site were disrupted. It delays further 3,4-DHBA binding at the active sites which lead to decrease the immobilized 3,4-POD kinetic rate. Nevertheless, a higher 3,4-DHBA RE (71%) was achieved using the immobilized 3,4-POD as compared with free counterpart (54%). Similar observations were obtained for other enzymes mediated biodegradation processes^55^,^56^. The only 7% RE of F-MWCNTs was observed which might be attributed to its adsorption behavior. It suggests negligible removal of 3,4-DHBA from wastewater by using the F-MWCNT alone. This further supported the desirable biodegradation effects of immobilized 3,4-POD towards the 3,4-DHBA removal (64%).

## Conclusions

The enzyme 3,4-POD was successfully immobilized onto well-dispersed F-MWCNT matrix for degrading the 3,4-DHBA pollutants in water. A maximum loading of 3,4-POD i.e. 1060 μg per mg of F-MWCNTs was achieved. Although 44% of the relative conformational changes of immobilized 3,4-POD was observed, >90% of relative activity and about 50% of catalytic efficiency were retained as compared with the free 3,4-POD. Immobilized 3,4-POD was less sensitive to higher alkaline pH and temperatures compared with its free counterparts. Higher shelf life of the immobilized 3,4-POD (>55% of residual activity on 30 days storage at 4 °C and 25 °C) could be an advantage for long-term storage and transportation in remote areas. Although the 3,4-DHBA binding affinity decreased towards the immobilized 3,4-POD, its higher recrudescence and reusability (>60% of residual activity after ten operational cycles) could compensate this gap, and thereby defraying the production cost of free 3,4-POD for long term uses in pollutant removal. Finally, combined RE of the immobilized 3,4-POD (71%) was also higher than the free 3,4-POD (54%), presenting a promising method to efficiently remove 3,4-DHBA from water.

## Materials and Methods

### Materials and Reagents

MWCNT of 12 ± 5 and 4 nm in outer and inner diameters, and >1 μm in length were purchased from the Bayer MaterialScience AG (Germany). The tubes were prepared by catalytic chemical vapor deposition which contained >95% carbon by weight. Pure 3,4-POD lyophilized powder (≥3 units/mg solid) from *Pseudomonas* sp. was purchased from the Sigma Aldrich and used without further purification. Sulfuric acid (98%), nitric acid (65%), hydrogen chloride (37%), sodium hydroxide, ethanol (70%), EDC, N-hydroxysuccinimide (NHS), 2-(*N*-morpholino)ethanesulfonic acid (MES), HEPES, 2-(Cyclohexylamino)ethanesulfonic acid (CHES), 4-(Cyclohexylamino)-1-butanesulfonic acid (CABS), Tween 20, 3,4-DHBA, Bradford, BCA, and bovine serum album (BSA) were purchased from the Sigma-Aldrich Sdn Bhd. (Malaysia). Deionized water (pH 6.8) was used in all of the experiments.

### Preparation of Functionalized Water Soluble MWCNTs

MWCNTs (0.5 g) were functionalized with 8.0 ml mixture of H_2_SO_4_ and HNO_3_ (3:1 v/v)^57^. The mixture was then sonicated at 50 °C for 8 h in an ultrasonication bath (Series 400, Powersonic, 40 KHz; Korea). All the F-MWCNTs were extracted from the residual acids, bases, metallic byproducts and carbonaceous impurities by repeated cycle of dilutions followed by centrifugations at 7000 rpm for 30 min (Beckman Coulter Allergra X-30R, USA). The supernatant was carefully decanted when the F-MWCNTs were precipitated at the bottom of the polyethylene centrifuge tube. The procedure was repeated 5–6 times until the resistivity of the supernatant was greater than 0.5 MΩ.cm, and pH was ~7.0. The F-MWCNTs were then rinsed with ethanol and dried overnight in a vacuum oven at 100 °C. Finally, the F-MWCNTs were stored in desiccators as dry powders for further uses. The pristine and F-MWCNTs functionalities were detected by ATR-IR spectroscopy (IFS 66 v/S, Bruker, Germany). In addition, TGA (TGA/SDTA 851, Mettler Toledo, USA) was used to check the pristine and F-MWCNT weight loss under air-flow (50 ml) from 25 to 1000 °C at 10 °C/min.

In order to determine the F-MWCNT solubility, 5.0 mg of its mixed into 5.0 mL of 50 mM HEPES buffer (pH: 8.0). The solution was then sonicated for 1 h to get a stable homogenous colloidal CNT suspension. After the solution was settled, supernatants of desired volumes were withdrawn at 0 and 30 days, and the concentrations of F-MWCNTs were measured spectrophotometrically at 500 nm (V-630, JASCO, Japan)^45^.

### Covalent Coupling of F-MWCNTs with 3,4-POD

The 3,4-POD was immobilized onto F-MWCNT surfaces using a two-step carbodiimide reaction^28^. Firstly, the F-MWCNTs (1.0 mg) were mixed into 1.0 mL MES buffer (50 mM, pH 5.95). The resulting solution was sonicated for 30 min in order to get a well-dispersed F-MWCNT solution. A 2.0 mL mixture of NHS (100 mM) and EDC (10 mM) solution was prepared using the same buffer and added into the 1.0 mL sonicated F-MWCNT solution. The final solution (3.0 mL) was then stirred at 400 rpm for 30 min at 25 ± 1 °C. The NHS/EDC activated F-MWCNTs were then centrifuged (15000 rpm, 10 min) to remove excess reactant and thoroughly rinsed with fresh MES (50 mM, pH 5.95) buffer solution. Secondly, the activated fresh F-MWCNTs were then transferred into a cold solution of 3,4-POD enzymes in HEPES (50 mM and pH 8.0). The effects of 3,4-POD concentrations (20, 40, 60, 100, 200, 500, 1000 and 2000 μg) were investigated to obtain maximum enzyme attachment and activity. The mixture was allowed to contact under continuous magnetic stirring (400 rpm) at 4 ± 2 °C. Different incubation times (1, 2, 4, 6 and 10 h) were studied for optimum 3,4-POD activity. Unbound 3,4-POD was removed by consecutive centrifugations and washing steps using fresh cold 50 mM HEPES buffer (pH 7.4). Washing protocols were continued (6–7 times) until no residual enzyme activity was detected in the washing solutions, and finally washed once with 0.5% Tween 20 in order to remove nonspecific bound enzymes. All collected washing solutions were analyzed for protein content using the BCA^58^ and Bradford^59^ assays. The BSA was used as standard for enzyme concentration assay.

The ATR-IR spectroscopy was performed for detecting the immobilized 3,4-POD onto F-MWCNT functionalities. Surface morphologies and topologies of the F-MWCNTs and immobilized 3,4-POD were determined using FE-SEM (Hitachi-SU8000, Japan) and TEM (Hitachi-HT7700, 120 kV, Japan). Herein both the F-MWCNTs and immobilized 3,4-POD were dispersed into fresh MilliQ water separately and mounted onto lacey copper grids for FE-SEM and TEM analyses. The AFM (BrukerBioScope Catalyst, Germany) images of the immobilized 3,4-POD on a glass substrate were collected using commercial silicon tips with a frequency range 51–94 kHz and analyzed by Nanoscope software. An UV-Vis spectrophotometer was used for detecting free and immobilized 3,4-POD in HEPES (50 mM, pH 7.4) solutions. The CD spectroscopy (J-810, Jasco, Japan) was performed to determine the structural changes of free and immobilized 3,4-POD. Test solution containing 0.05 mg/ml of either free or immobilized 3,4-POD in CHES buffer (10 mM, pH 5.6) was prepared from which 300 μL was used for CD analyses. A solution of F-MWCNT (15 μg/ml) equivalent with the concentration of immobilized 3,4-POD support was used as control in the same buffer solution. All readings were obtained from three consecutive scans for each CD spectra.

### Free and Immobilized 3,4-POD Activity Assays

The free and immobilized 3,4-POD activities were determined spectrophotometrically from the disappearance of 3,4-DHBA with time at 290 nm (ε_290 nm_: 3890 M^−1^ cm^−1^)^14^,^18^. The reaction cuvette contained 100 μM of 3,4-DHBA, suitable amounts of free and immobilized 3,4-POD and buffer to bring the total volume of 3.0 mL. Optimum pH of the free and immobilized 3,4-POD was determined using 50 mM of MES (pH 4.0–7.0), HEPES (7.5–8.5), CHES (8.5–10.0), and CABS (10.5–11.0) at 30 °C. Temperatures range from 5 °C to 80 °C was studied at 50 mM CHES and optimum pH 9.0 and 10.0 for the free and immobilized 3,4-POD activities, respectively. A constant stirring speed was used to ensure through mixing during the assay. One unit of activity was defined as the amount of enzyme required to oxidize 1 *μ*moL of 3,4-DHBA per minute. Immobilized 3,4-POD activity was expressed as activity unit (*U*_x_) per milligram of F-MWCNTs (*W*_MWCNT_), where *U*_X_ is the activity (unit) of the immobilized 3,4-POD assayed by similar free enzyme method as eq. (3)


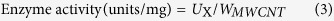


The kinetic parameters (*V*_*max*_, *K*_*m*_, *K*_*cat*_ and *K*_*cat*_*/K*_*m*_) of the free and immobilized 3,4-POD were determined from the non-linear regression of Michaelis-Menten model^60^ with 3,4-DHBA concentrations (1–200 μM) at 50 mM CHES, optimum pH 9.0 and 10.0 with optimum temperatures 55 °C and 60 °C; respectively.

### Free and Immobilized 3,4-POD Stability Assays

First, thermostabilities of the free and immobilized 3,4-POD were checked at 90 °C. Aliquots were withdrawn at regular time intervals for assaying the residual 3,4-POD activities. Second, the storage stability of the free and immobilized 3,4-POD was determined at 4 °C and 25 °C for one month. The residual activities of free and immobilized 3,4-POD were measured at regular day intervals. At last, recycling of the immobilized 3,4-POD was performed according to the following method: 300 μL of the immobilized 3,4-POD onto F-MWCNTs (1 mg/mL) was mixed into 100 μM of 3,4-DHBA, and the activities were assayed as mentioned earlier. The immobilized 3,4-POD onto FMWCNTs was then recovered by centrifugation (14000 rpm, 10 min), and washed five times with fresh CHES buffer (50 mM, pH 10.0) in order to remove the residual 3,4-DHBA and its oxidized products. A total of ten cycles was performed, and the results were expressed as an average of three replicates.

### Removal of 3,4-DHBA

Batch experiments were performed in 10.0 mL screw tabs sealable glass reaction bottles containing 2.0 mg of the F-MWCNTs as control, free, and immobilized 3,4-POD in CHES (50 mM, pH 9.0). All of these samples were treated with the 5.0 mM of 3,4 DHBA solutions. The reaction mixtures were stirred at 200 rpm, 25 ± 1 °C. Aliquots were withdrawn at regular time intervals for measuring the absorbance of 3,4-DHBA at 290 nm^18^. The amount of 3,4-DHBA biodegraded by the immobilized 3,4-POD was calculated using eq. (4):


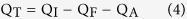


where *Q*_T_ is the amount of 3,4-DHBA (mM) degraded by the immobilized 3,4-POD, *Q*_I_ is the initial 3,4-DHBA concentration (mM) in the solution, *Q*_F_ is the amount of 3,4-DHBA (mM) retained in the solution and *Q*_A_ is the 3,4-DHBAconcentration (mM) adsorbed onto F-MWCNTs. All treatments were replicated five times, and the average values were calculated.

## Additional Information

**How to cite this article**: Das, R. *et al.* Highly Efficient and Stable Novel NanoBiohybrid Catalyst to Avert 3,4-Dihydroxybenzoic Acid Pollutant in Water. *Sci. Rep.* **6**, 33572; doi: 10.1038/srep33572 (2016).

## Supplementary Material

Supplementary Information

## Figures and Tables

**Table 1:** Relative Activity and Kinetic Parameters of Free and Immobilized 3,4-POD.

3,4-POD	Relative Activity^a^ (%)	*V*_*max*_ (μmole/min)	*K*_*m*_ (μM)	*K*_*cat*_ (min^−1^)	*K*_*cat*_*/K*_*m*_ (min^−1^.μM^−1^)
Free	—	153 ± 24	37 ± 15	210 ± 33	5.61 ± 3.0
Immobilized	>90	189 ± 69	95 ± 71	258 ± 94	2.71 ± 2.0

^a^The relative activity was determined by (Specific activity of immobilized 3,4-POD/Specific activity of free 3,4 POD) × 100.

**Table 2:** Kinetic Parameters and Removal Efficiency of the Free and Immobilized 3,4-POD Toward 3,4-DHBA.

Sample	*k*^−1^ (h)	t_*1/2*_ (h)	RE_6_ (%)
Removal of F-MWCNTs Alone	0.04	1.71	7
Total Removal by Immobilized 3,4-POD	0.18	3.81	71
Degradation by Immobilized 3,4-POD	0.16	4.26	64
Free 3,4-POD	1.85	0.37	54
