# Supplementary material for: Highly Efficient and Stable Novel NanoBiohybrid Catalyst to Avert 3,4-Dihydroxybenzoic Acid Pollutant in Water
Source: Sci Rep. 2016 Oct 10;6:33572. doi: 10.1038/srep33572 (PMC5056344; doi:10.1038/srep33572)
Supplement: Supplementary Information [file srep33572-s1.pdf]

Appendix A

Supporting Information

Highly Efficient and Stable Novel NanoBiohybrid Catalyst to Avert 3,4-Dihydroxybenzoic Acid Pollutant in Water

Rasel Das[1,*], Sharifah Bee Abd Hamid[1,*] & Mohamad Suffian Mohamad Annuar[2]

[1]Nanotechnology and Catalysis Research Center (NANOCAT), University of Malaya, 50603 Kuala Lumpur, Malaysia.

[2]Institute of Biological Sciences, Faculty of Science, University of Malaya, 50603 Kuala Lumpur, Malaysia.

*Corresponding Author: Tel: + (60) 03-7967-6959; Fax: + (60) 03-7967-6956; E-mail:

raselgeneticist@gmail.com (R.D.) & sharifahbee@um.edu.my (S.B.A.H)

Appendix A

UV-Vis observations of linear superimposible relationships between the F-MWCNT absorbances *versus* concentrations

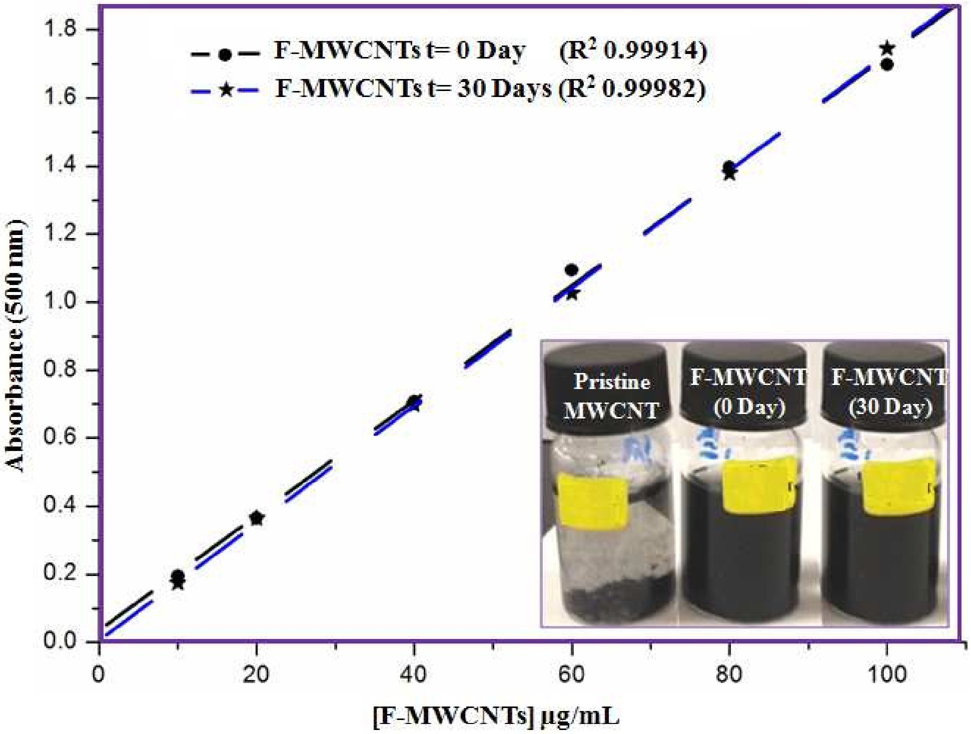

Figure S1: Absorbance of F-MWCNT concentrations as a function of time (Herein: (•) freshly prepared (t = 0) and (*) after one month of storage (t = 30 days). As shown in Figure (inset), the camera observation showed the pristine MWCNT was coagulated and precipitated at the bottom of the vial upon sonication. In contrast, a greater solubility and black homogenous F-MWCNT solution was pertained for one month supporting the UV-Vis data.

Appendix A

Effect of time incubations on immobilized 3,4-POD activities

Figure S2 shows only 2 h incubation of 3,4-POD attachment was sufficient to obtain saturated immobilized 3,4-POD activities.

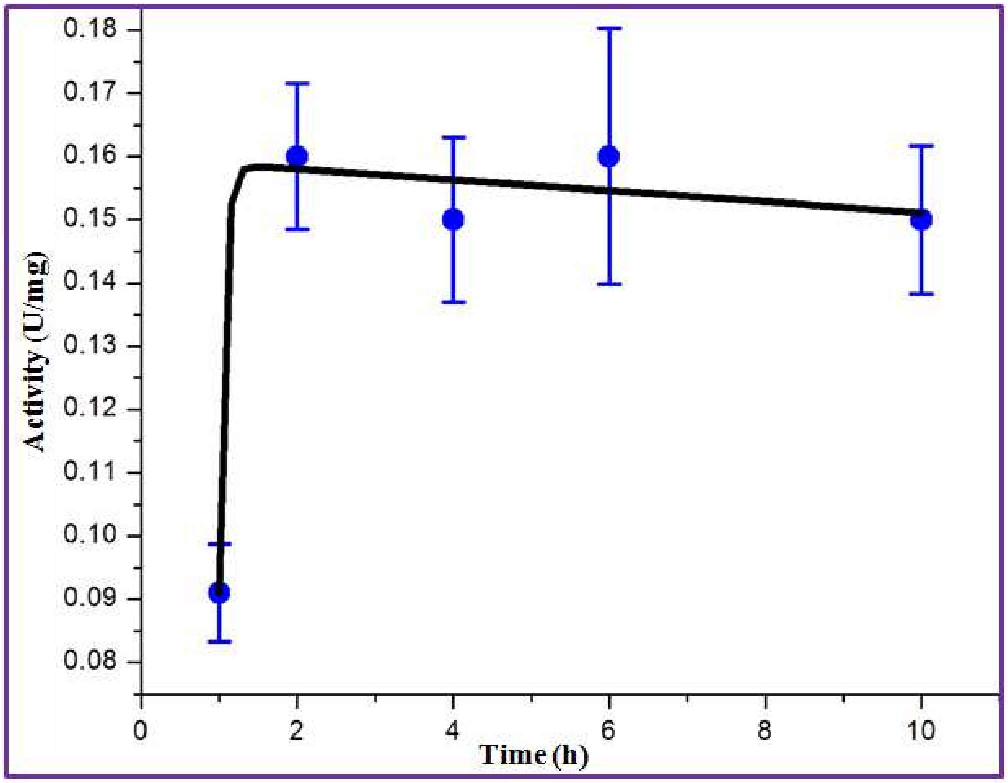

Figure S2: Activity of immobilized 3,4-POD at different time incubations of reactions between the 3,4-POD and the F-MWCNTs.

Appendix A

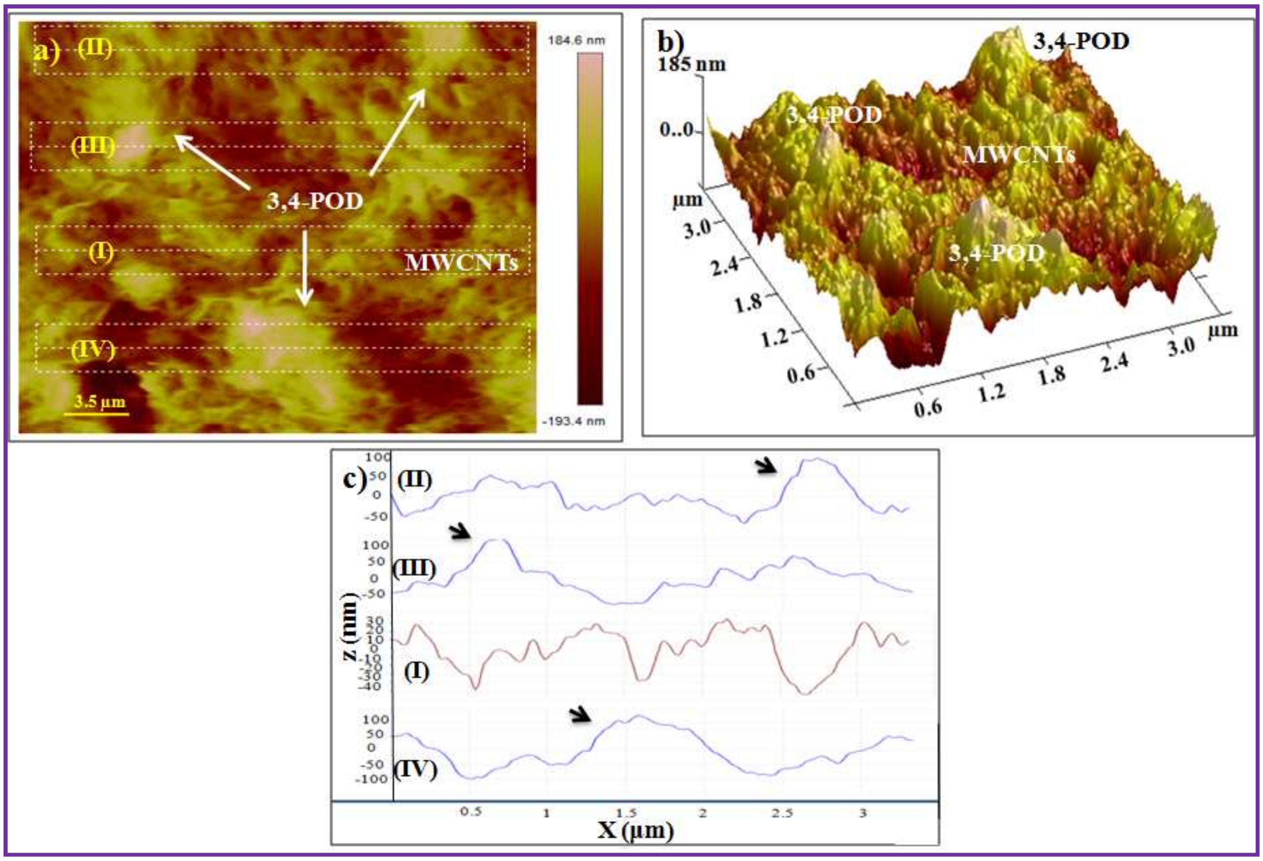

Figure S3: AFM images of the immobilized 3,4-POD: (a) bidimensional (b) tridimensional analyses, herein dotted lines from I to IV in panel (a) were the sections for further analyses as shown in image profiles (c) : blacked arrows indicate peaked sizes of the immobilized 3,4-POD.

Appendix A

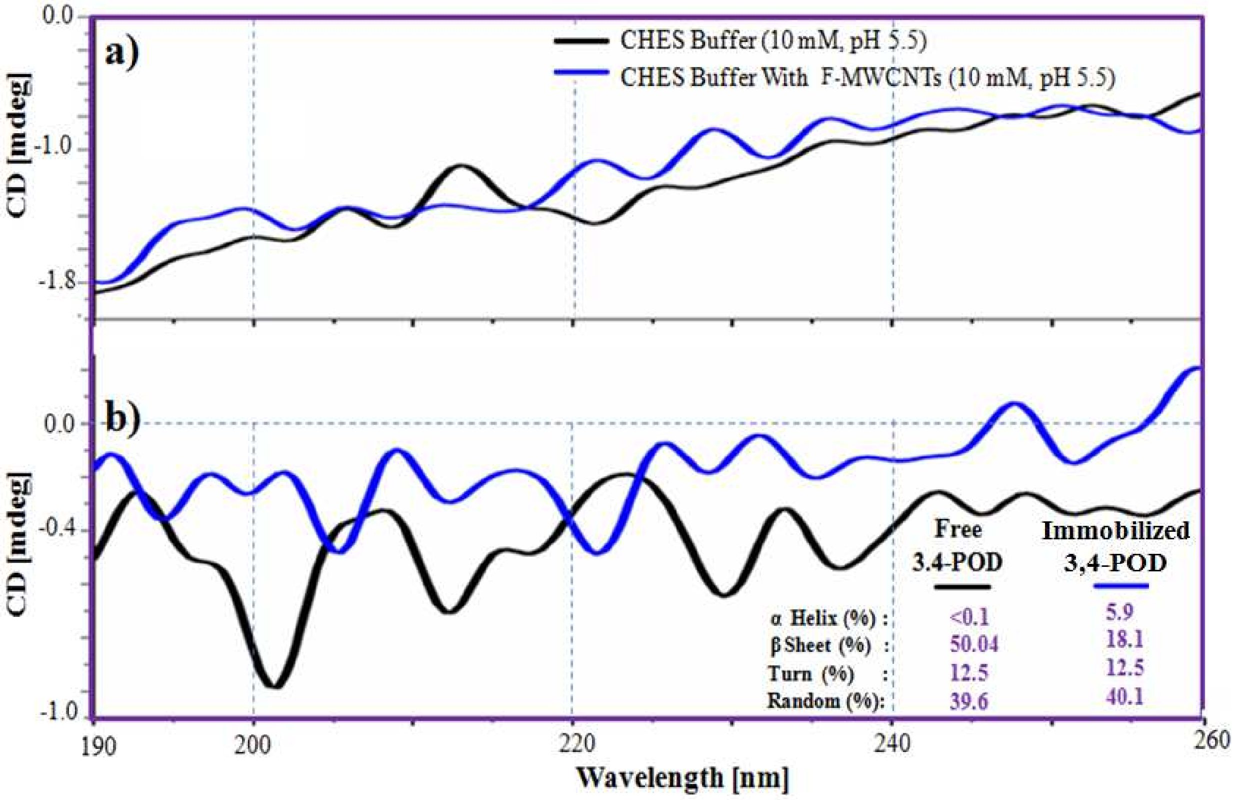

Figure S4: CD spectra of (a) controls and (b) sample solutions of the free and immobilized 3,4-POD in the Far-UV region. CD spectra of CHES buffer and the F-MWCNT solutions controls (a); and the free and immobilized 3,4-POD (b). As shown in (a), no significant differences were observed in the entire wavelengths of CHES and F-MWCNT spectra. On the other hand, (b) postulates the differences in secondary structures (%) such as α-helices, β-sheets, turns and random coils of the free and immobilized 3,4-POD. The mean residue ellipticity (θ_{218}) was used to measure relative structural changes (%) of the free and immobilized 3,4-POD.

Appendix A

Michaelis-Menten nonlinear regression analyses of the free and immobilized 3,4-POD activities as shown in Figures S5 and S6, respectively.

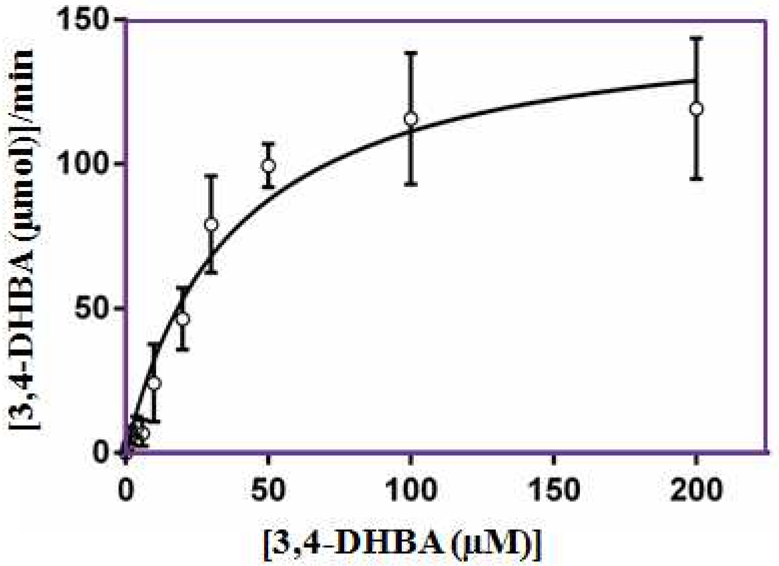

Figure S5: Michaelis-Menten plot of the free 3,4-POD.

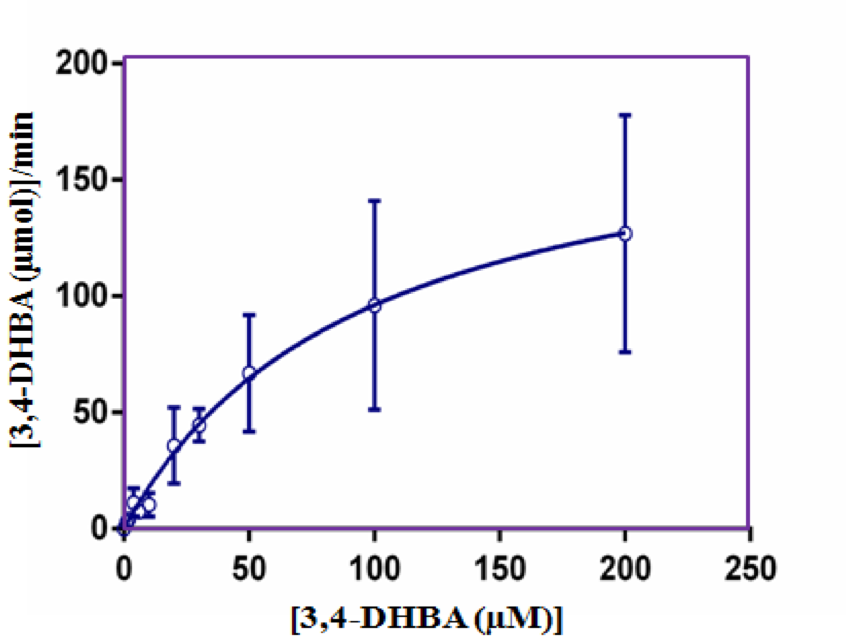

Figure S6: Michaelis-Menten plot of the immobilized 3,4-POD.
